# Miniature Microwave Notch Filters and Comparators Based on Transmission Lines Loaded with Stepped Impedance Resonators (SIRs)

**DOI:** 10.3390/mi7010001

**Published:** 2015-12-23

**Authors:** Lijuan Su, Jordi Naqui, Javier Mata-Contreras, Ferran Martín

**Affiliations:** Centre d’Investigació en Metamaterials per a la Innovació en Tecnologies Electrònica i de Comunicacions (CIMITEC), Departament d’Enginyeria Electrònica, Universitat Autònoma de Barcelona, 08193 Bellaterra (Barcelona), Spain; jordi.naqui@uab.cat (J.N.); franciscojavier.mata@uab.cat (J.M.-C.); ferran.martin@uab.cat (F.M.)

**Keywords:** stepped impedance resonator (SIR), microstrip lines, coplanar waveguides, microwave notch filter, microwave comparator

## Abstract

In this paper, different configurations of transmission lines loaded with stepped impedance resonators (SIRs) are reviewed. This includes microstrip lines loaded with pairs of SIRs, and coplanar waveguides (CPW) loaded with multi-section SIRs. Due to the high electric coupling between the line and the resonant elements, the structures are electrically small, *i.e.*, dimensions are small as compared to the wavelength at the fundamental resonance. The circuit models describing these structures are discussed and validated, and the potential applications as notch filters and comparators are highlighted.

## 1. Introduction

**Figure 1:**
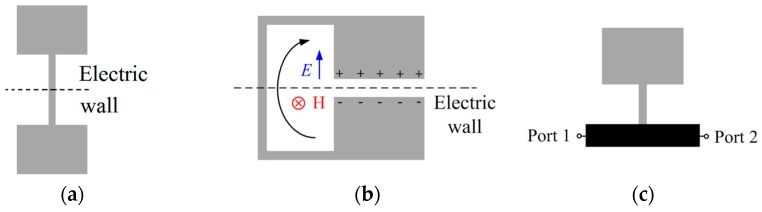
Typical topology of a tri-section stepped impedance resonators (SIR) (**a**), folded-SIR (**b**) and stepped impedance shunt stubs (SISS) attached to a microstrip line (depicted in black color) (**c**). The symmetry planes (for **a** and **b**), corresponding to an electric wall at the fundamental resonance, are indicated.

Stepped impedance resonators (SIRs) were proposed in the late 1970s as electrically small semi-lumped (planar) resonant elements useful for the realization of microwave filters [1,2,3]. These resonators are typically (although not exclusively) implemented by means of a tri-section structure where a narrow strip (high impedance section) is sandwiched between two wide (and hence low impedance) sections. The typical topology is depicted in Figure 1a, whereas Figure 1b shows the topology of the folded-SIR. At the fundamental resonance, both topologies exhibit an electric wall at the bi-section plane of the resonator (indicated in the figures), and there is an electric dipole moment orthogonal to this plane at such resonance frequency. Thus, both structures can be excited by means of a time-varying electric field, with a non-negligible component in the direction of the electric dipole moment. The folded-SIR, however, can be driven not only electrically, but also by means of a time-varying magnetic field applied orthogonal to the plane of the resonator, since there is also a magnetic dipole moment in that direction [4]. Folded SIRs are electrically small and can be useful as an alternative to split ring resonators (SRRs) [5] for the implementation of negative effective permeability metamaterials [4]. SIRs (including meandered SIRs and multi-section SIRs) and folded-SIRs have found numerous applications in microwave engineering, where size reduction has been a due [1,2,3,6,7].

In most of the previous applications, the resonators are coupled or attached to a host transmission line. A high level of miniaturization has been achieved in SIR-based coplanar waveguide (CPW) structures, where elliptic filters [6] and radiofrequency (RF) barcodes (or spectral signature barcodes) [7] have been demonstrated. In microstrip technology, the so-called stepped impedance shunt stub (SISS), consisting on half of the structure of Figure 1a directly in contact with a host microstrip line (Figure 1c), can be used for the implementation of notch filters, and a detailed analysis is reported in [8]. To a first order approximation the SISS can be modeled as a shunt connected series LC (inductance and capacitance) resonator.

In this paper, we will review some of the applications of transmission lines loaded with SIRs. Specifically, the focus is on asymmetric structures, or symmetric structures that can be made asymmetric by appropriately loading the SIRs. We will consider both microstrip and CPW transmission lines loaded with SIRs, and the applications include dual-band microwave notch filters and comparators (*i.e.*, structures able to detect defects or abnormalities in samples, as compared to a reference). In Section 2, the microstrip line loaded with a pair of SISS is analyzed and modeled, and the model is validated experimentally. Section 3 deals with SIR-loaded CPWs, where two different structures are considered: A 5-section SIR-loaded CPW, where the central (wide) section of the 5-SIR is capacitively coupled to the CPW, and the same structure (5-SIR) but directly connected to the central strip of the CPW through metallic vias. In both cases, the 5-SIR is etched in the back substrate side of the CPW transmission line. The models of both structures are also presented and validated. In Section 4, the applicability of these SIR-based structures to dual-band notch filters and comparators is demonstrated. Finally, the main conclusions are highlighted in Section 5.

## 2. Microstrip Line Loaded with Pairs of SISSs

**Figure 2:**
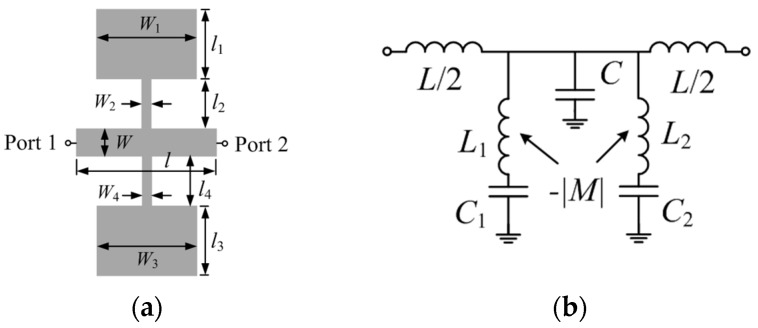
(**a**) Microstrip line section loaded with a pair of SISS; (**b**) lumped element equivalent circuit model.

Figure 2a depicts a microstrip line section loaded with a pair of SISS. Assuming that the microstrip line is electrically short, and that there is a high impedance contrast between the narrow and wide sections of the SISS, the structure can be described by the lumped element equivalent circuit shown in Figure 2b [9]. The model considers the general case of an asymmetric structure, where the SISS are modeled by the inductances *L*_1,2_ and the capacitances *C*_1,2_, and the microstrip line section is accounted for by the capacitance *C* and the inductance *L.* The coupling (magnetic) between the two SISS cannot be neglected and is modeled through the mutual inductance *M* (such coupling is negative because the currents in the inductances flow in opposite directions). Losses are not considered in the model.

The transmission zeros of the structure are given by those frequencies that null the reactance of the series branch, that is:

(1) $$\omega_{\pm }^{2}=\frac{A\pm \sqrt{B}}{D}$$

with

$$A=L_{1}C_{1}+L_{2}C_{2}$$

$$B={\left(L_{1}C_{1}-L_{2}C_{2}\right)}^{2}+4C_{1}C_{2}M^{2}$$

$$D=2C_{1}C_{2}(L_{1}L_{2}-M^{2})$$

**Figure 3:**
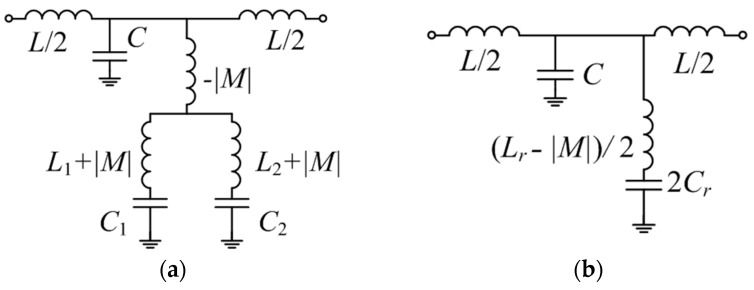
(**a**) Equivalent circuit model to that of Figure 2b; (**b**) Equivalent circuit model for the symmetric case.

Expression Equation (1) can be easily inferred from the transformed model of Figure 2b, depicted in Figure 3a. If the structure is symmetric (*i.e*., *L*_1_ = *L*_2_ ≡ *L_r_* and *C*_1_ = *C*_2_ ≡ *C_r_*), the mathematical solutions of Equation (1) are:

(2) $$\omega_{\pm }=\frac{1}{\sqrt{(L_{r}\mp \left|M\right|)\cdot C_{r}}}$$

However, ω_−_ is not actually a physical solution since it nulls the denominator of the reactance (obviously, for the symmetric case only one notch at the fundamental frequency of the SISS is expected, as results from the circuit model depicted in Figure 3b). Thus, the mutual coupling between the two inductors of two SISSs has the effect of increasing the notch frequency (symmetric case).

**Figure 4:**
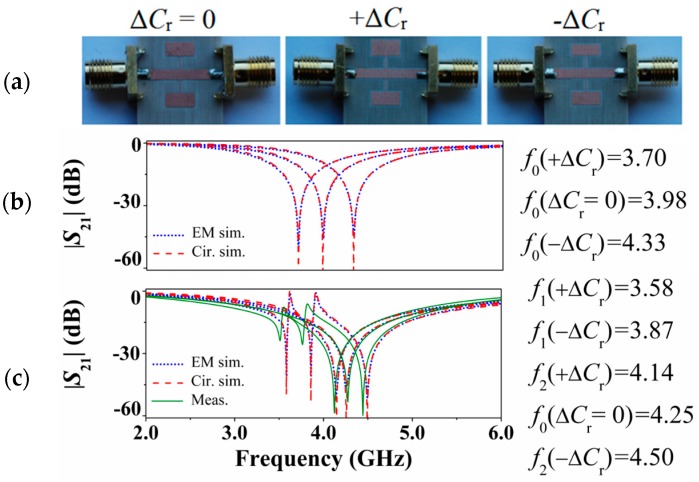
(**a**) Photograph of the fabricated SISS-loaded transmission lines, and transmission coefficient corresponding to the lossless electromagnetic and circuit simulations for a microstrip line loaded (**b**) with a single SISS (not fabricated), and (**c**) with a pair of SISSs (fabricated and shown in Figure 4a). In (**c**), measurements are included. The dimensions (in reference to Figure 2a) are *W* = 1.83 mm, *l* = 15.9 mm, *l*_1_ = *l*_2_ = *l*_3_ = *l*_4_ = 2.6 mm, Δ*l*_1_ = ±0.5 mm, *W*_1_ = *W*_3_ = 5.5 mm, *W*_2_ = *W*_4_ = 250 µm. The substrate is *Rogers RO4003C* with considered dielectric constant ε*_r_* = 3.1, thickness *h* = 812.8 µm, and loss tangent tan*δ* = 0.0021. The circuit values are *L* = 1.80 nH, *C* = 0.57 pF, *L_r_* =2.46 nH, *C_r_* = 0.65 pF, ±Δ*C*_r_ = ±0.15*C_r_* = ±0.10 pF, and *M* = −0.31 nH. Reprinted with permission from [9].

The validation of the model has been done by comparing full wave electromagnetic simulations with circuit simulations with extracted parameters of different structures. To extract the parameters, we have first considered microstrip lines loaded with a single SISS, following a procedure reported in [9], and similar to that reported in [10]. Then *M* has been obtained in the structures loaded with pairs of SISS by curve fitting. The agreement is good, as depicted in Figure 4, where the responses of three different structures are presented. One of such structures is symmetric, whereas the other two are obtained from the first one by increasing or decreasing one of the capacitances, as indicated. The responses of the microstrip lines loaded with single SISS are also indicated, so that the positive shift of the transmission zero for the symmetric structure can be appreciated. Note also that the agreement with the responses of the fabricated structures is also good (except by the effect of losses, not considered in the model, and fabrication related tolerances).

## 3. CPW Loaded with 5-SIRs

**Figure 5:**
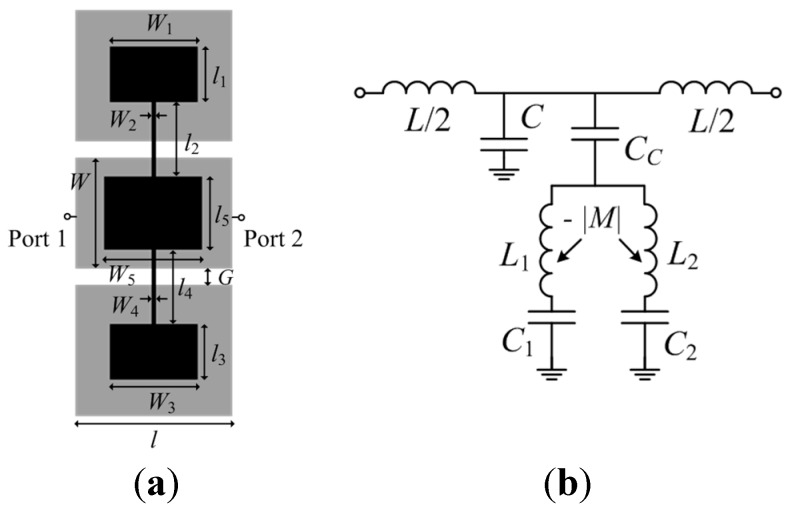
(**a**) Coplanar waveguide (CPW) loaded with a 5S-SIR and (**b**) circuit model. Relevant dimensions are indicated.

Figure 5a depicts a CPW line section loaded with a 5S-SIR, etched in the back substrate side. The equivalent circuit model is depicted in Figure 5b [11], where *L* and *C* are the inductance and capacitance of the CPW line section, and *L*_1,2_ and *C*_1,2_ describe the inductances and capacitances of the middle and external sections, respectively, of the 5S-SIR. The 5-SIR is electrically coupled to the line through *C_c_*, the broadside capacitance between the central strip of the CPW and the central section of the 5S-SIR. Finally, the magnetic coupling between the two inductances of the resonator is accounted for by *M* (negative, for the reasons explained in reference to the SISS-loaded microstrip line of the previous section). Since the considered structure is electrically short, it is reasonable to assume, to a first order approximation, that the slot mode is not generated (the ports in the electromagnetic simulation and the connectors in the measurement act as air bridges, effectively connecting the two ground plane regions).

In this case, the transmission zero frequencies are given by Equation (1) with:

$$A=C_{1}C_{2}(L_{1}+L_{2}-2M)+C_{c}(L_{1}C_{1}+L_{2}C_{2})$$

$$B=C_{1}^{2}C_{2}^{2}{\left(L_{1}+L_{2}-2M\right)}^{2}+C_{c}^{2}{\left(L_{1}C_{1}-L_{2}C_{2}\right)}^{2}+2C_{c}C_{1}C_{2}\times \left\{L_{1}C_{1}(L_{1}-L_{2}-2M)+L_{2}C_{2}(L_{2}-L_{1}-2M)+2M^{2}(C_{1}+C_{2}+C_{c})\right\}$$

$$D=2C_{c}C_{1}C_{2}(L_{1}L_{2}-M^{2})$$

If the structure is symmetric (*i.e.*, *L*_1_ = *L*_2_ ≡ *L_r_* and *C*_1_ = *C*_2_ ≡ *C_r_*), the mathematical solutions are of the form:

(3a) $$\omega_{+}=\frac{1}{\sqrt{(L_{r}-\left|M\right|)\cdot \frac{C_{c}C_{r}}{C_{c}+2C_{r}}}}$$

(3b) $$\omega_{-}=\frac{1}{\sqrt{(L_{r}+\left|M\right|)\cdot C_{r}}}$$

However, ω_−_ is not actually a physical solution since it nulls the denominator of the reactance. Thus, the mutual coupling between the two inductors of the two 5-SIR has the effect of increasing the notch frequency for the symmetric case, *i.e.*, a behavior identical to the one of the microstrip line loaded with a pair of SISS.

**Figure 6:**
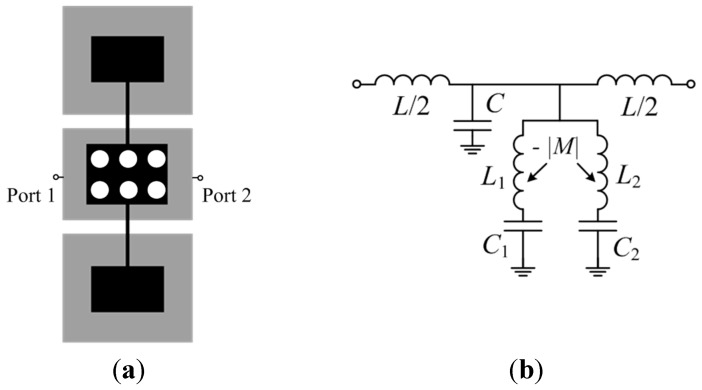
(**a**) CPW loaded with a 5S-SIR directly in contact to the central strip of the CPW through metallic vias, and (**b**) circuit model.

A variation of the previous CPW structure consists of a direct connection (through vias) of the 5-SIR to the central strip of the CPW, as depicted in Figure 6. This effectively shorts the capacitance *C_c_*, and the resulting circuit model is identical to the one depicted in Figure 2b.

The validation of the models of these CPW loaded structures has been also carried out by comparison between the frequency responses inferred from full wave electromagnetic simulation and the responses derived from circuit simulation with the parameters conveniently extracted. For the structure of Figure 5a the parameter extraction method is more complex (as compared to the one of the previous section) since we have an additional parameter, namely, *C_c_* (the details can be found in [11]). Indeed, the procedure first considers the structure with vias, so that all the parameters, except *C_c_*, are determined; then *C_c_* is determined by curve fitting.

**Figure 7:**
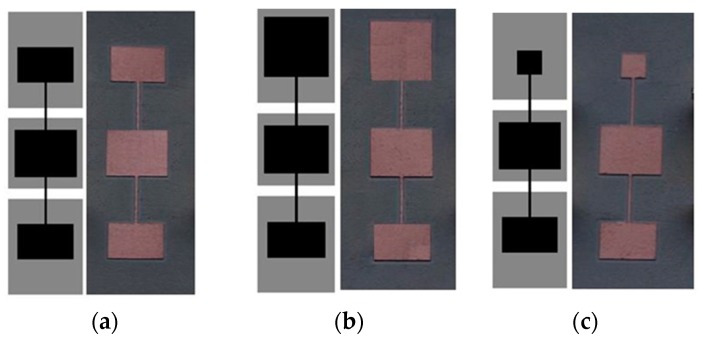
Considered 5S-SIR-loaded CPWs: (**a**) symmetric; (**b**) asymmetric with one capacitance larger; (**c**) asymmetric with one capacitance smaller. Dimensions are (in reference to Figure 5): for CPW lines: *l* = 4.5 mm, *W =* 6 mm and *G=* 0.96 mm*,* corresponding to 50 Ω. For 5S-SIR: (**a**) *W*_1_ = 4.5 mm, *l*_1_ = 3 mm, *W*_3_ = 4.5 mm, *l*_3_ = 3 mm, *W*_2_ = *W*_4_ = 0.2 mm, *l*_2_ = *l*_4_ = 4 mm, *W*_5_ = 5 mm, *l*_5_ = 4 mm. For (**b**) *W*_1_ = *l*_1_ = 5 mm and for (**c**) *W*_1_ = *l*_1_ = 2 mm, with the other dimensions the same as in (**a**). The considered substrate (*Rogers RO3010*) has thickness of *h* = 0.635 mm and dielectric constant of *ε_r_* = 11.2.

**Figure 8:**
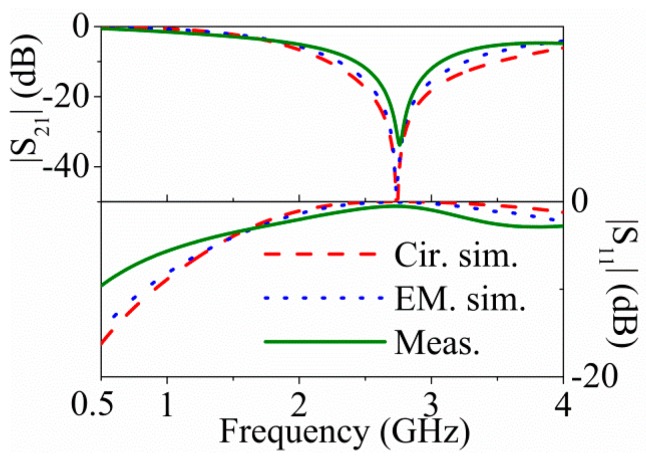
Electromagnetic simulation, circuit simulation and measurement response for the symmetric structure of Figure 7a. Reprinted with permission from [11].

The three considered structures: For model validation are depicted in Figure 7 (dimensions and substrate parameters are indicated in the caption). One is symmetric and the other two asymmetric, where the two asymmetric structures are derived from the symmetric one by increasing or decreasing the area of one of the external patch capacitors, while the other external patch capacitors for these two asymmetric structures keeps the same dimensions as in the symmetric one. The element values of the circuit model for the symmetric structure are *L* = 3.49 nH, *C* = 1.21 pF, *L_r_* = 4.20 nH, *C_r_* = 2.65 pF, *M* = −1.08 nH, and *C_c_* = 3.62 pF. The comparison of the electromagnetic simulation (using Keysight Technologies Momentum, Keysight Technologies Inc.，Santa Rosa, CA, USA) and circuit simulation of the symmetric structure is shown in Figure 8 (the measurement data is included as well), where good agreement can be appreciated, pointing out the validity of the proposed model.

**Figure 9:**
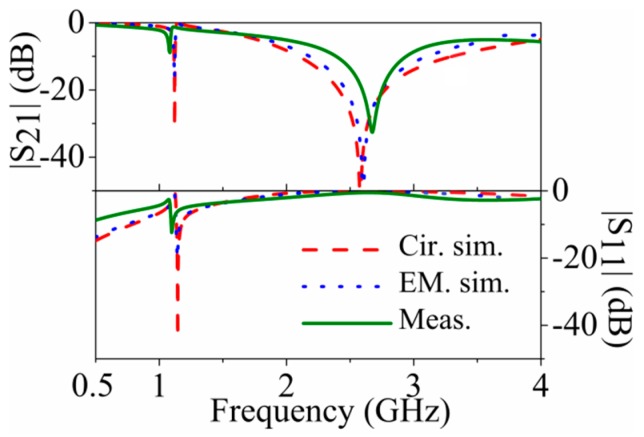
Electromagnetic simulation, circuit simulation and measurement response for the asymmetric structure of Figure 7b. Reprinted with permission from [11].

**Figure 10:**
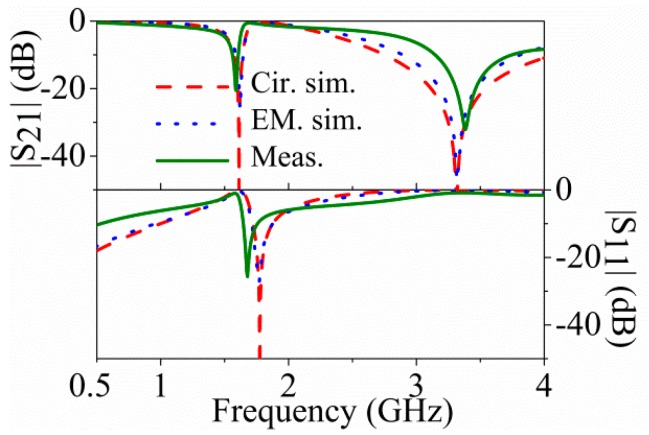
Electromagnetic simulation, circuit simulation and measurement response for the asymmetric structure of Figure 7c. Reprinted with permission from [11].

For the asymmetric cases, the small external patch inductance and capacitance of the 5-SIR have been found to be 4.30 nH and 0.97 pF, and the big external patch inductance and capacitance of 5-SIR have been found to be 4.26 nH and 4.53 pF, whereas the mutual inductances for these two cases have been found to be −1.19 nH and −1.06 nH respectively, *i.e.*, very similar values, and also similar to the value corresponding to the symmetric structure. This indicates that *M* is scarcely dependent on the dimensions of the patch capacitances of the 5S-SIR, as expected. The resulting middle patch capacitances of small and big structures are 3.61 pF and 3.75 pF respectively. The agreement between the electromagnetic simulation, circuit simulation and measurement for the two asymmetric cases (Figure 9 and Figure 10) is reasonable.

**Figure 11:**
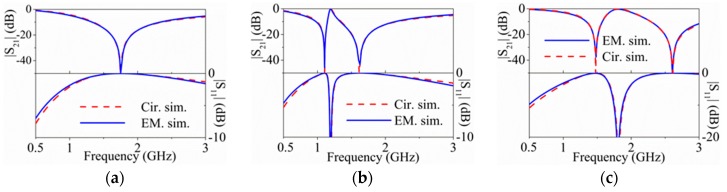
Electromagnetic simulation, and circuit simulation of the structures of Figure 7 with vias connecting the central patch of the 5-SIR and the central strip of the CPW transmission line: (**a**) symmetric; (**b**) asymmetric with one capacitance larger; (**c**) asymmetric with one capacitance smaller.

For the structures with vias, good agreement between circuit and electromagnetic simulation has been also obtained, as Figure 11 reveals (these structures have not been fabricated).

## 4. Application to Microwave Notch Filters and Comparators

According to the results of the previous subsections, the symmetric structures can be used as single broadband notch filters. Dual-band functionality is achieved by asymmetrically loading the line, and expression Equation (1) can be used to set the position of the notches. Note that the SISS-loaded microstrip line and the 5-SIR-loaded CPW without vias exhibit a wideband notch and a very narrow notch, whereas for the 5-SIR-loaded CPW with vias, the width of both notches is comparable. The reason is that in the CPW with vias there is not a coupling capacitance (*C_c_*) in the shunt branch, and this favors sensitivity (also influenced by the mutual inductance, *M*). Thus, depending on the application (*i.e*., notch width requirement), one structure or the other may be more convenient.

**Figure 12:**
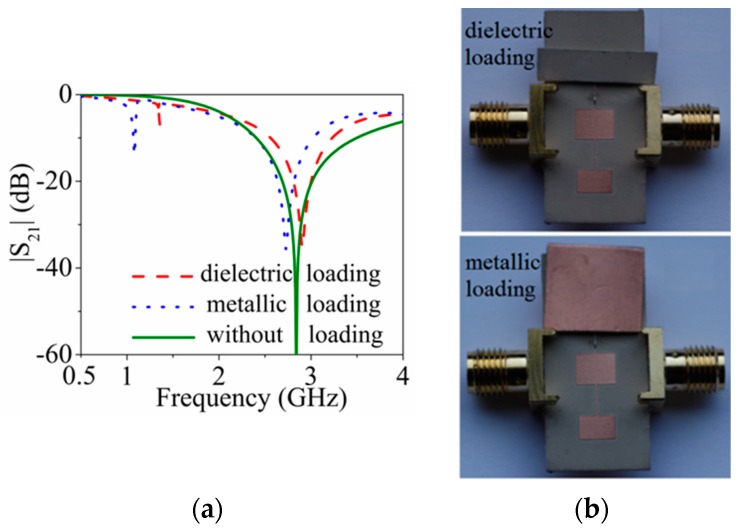
(**a**) Measured responses of the structure of Figure 7a with asymmetric dielectric loading and metallic loading; (**b**) fabricated prototypes loaded with dielectric loading and metallic loading, respectively. Reprinted with permission from [11].

In order to use the structures as microwave comparators, the SIR or SISS loaded lines must be symmetric. If line loading (dielectric or metallic) is symmetric, then the structure is expected to exhibit a single notch in the frequency response, whereas if the loading is asymmetric, two notches separated a distance depending on the level of asymmetry are expected. Thus, the reported structures are useful to determine differences between a sample under test (SUT) and a reference sample (*i.e.,* compare the two samples). To demonstrate the potential of these structures as comparators, the symmetric structure of Figure 7a has been loaded with a dielectric load (consisting of a small piece of Rogers RO3010 substrate with the copper removed from both substrate sides) placed on top of one of the patch capacitances. The measured response, shown in Figure 12, exhibits two notches, indicative of the asymmetric loading. Then, we have repeated the experiment by using the same piece of substrate but keeping the metal layers (metallic loading). The measured response is also included in Figure 12, where it can be seen that the depth of the first notch is superior (as compared to dielectric loading), since the structure is more sensitive to the effects of a metallic layer placed on top of one of the patch capacitances. The reason is that adding a metal increases more effectively the capacitance of the patch, as compared to dielectric loading.

As sensors, the SIR-based structures discussed in this paper belong to the category of resonance frequency splitting sensors. However, there is also another type of sensing structures based on symmetry properties: Coupling modulated resonance based sensors [12]. In this case, the sensor is based on a transmission line loaded with a single (symmetric) resonant element, the symmetry plane of the line and resonator are aligned, and these planes are of different electromagnetic nature. One of them is a magnetic wall, and the other one is an electric wall. Under these conditions, the resonator is not coupled to the line. However, by truncating symmetry, line to resonator coupling arises, producing a notch in the transmission coefficient, and the depth of this notch depends on the level of asymmetry, since it determines the coupling level. Several sensing structures based on these principles have been proposed (several of them by the authors) [13,14,15,16,17]. For instance, split ring resonant elements or complementary split rings have been used for sensing purposes. By using SIRs, the sensors are small (this extends also to notch filters) since the coupling with the host line is broadside. This is the main advantage over other sensors of this type based on other resonant elements. Also, ground plane etching is avoided (contrary to sensors based on complementary resonant elements).

Concerning the demand of SIR based sensors or comparators in microwaves, applications include sensors for dielectric characterization, quality control, and microfluidics, among others. In the paper, proof-of-concept demonstrators are presented. Since the electric field below the patches is high, significant sensitivity can be potentially achieved by using multilayer structures and arrangement of the structures under test in those regions.

## 5. Conclusions

In summary, it has been shown that miniature microwave notch filters and comparators can be implemented by means of transmission lines loaded with stepped impedance resonators (SIRs), including stepped impedance shunt stubs (SISS) in microstrip technology, and 5-section SIRs (5-SIRs) in coplanar waveguide (CPW) technology. The lumped element equivalent circuit models of these electrically small planar structures have been proposed and validated, and an analysis that has lead us to find the position of the transmission zero frequencies has been carried out. Finally, a proof of concept of microwave comparators has been presented.
